# Experience with 2 years’ intervention to progressively reduce salt supply to kitchens in elderly care facilities—challenges and further research: post hoc analysis of the DECIDE-Salt randomized clinical trial

**DOI:** 10.1186/s12916-023-03130-z

**Published:** 2023-11-03

**Authors:** Yifang Yuan, Aoming Jin, Peifen Duan, La’e Cao, Hongxia Wang, Senke Hu, Jiayu Li, Xiangxian Feng, Qianku Qiao, Hui Zhang, Ruijuan Zhang, Huijuan Li, Pei Gao, Gaoqiang Xie, Jianhui Yuan, Lili Cheng, Sujuan Wang, Wenyi Niu, Paul Elliott, Runlin Gao, Darwin Labarthe, Yangfeng Wu

**Affiliations:** 1 Department of Epidemiology and Biostatistics, Peking University School of Public Health, Beijing, China; 2 Peking University Clinical Research Center, Peking University First Hospital, Haidian District, 38 Xueyuan Road, Beijing, China; 3 Present Address: China National Clinical Research Center for Neurological Diseases, Beijing Tiantan Hospital, Capital Medical University, Beijing, China; 4 Changzhi Medical College, Shanxi, China; 5 Yangcheng Ophthalmic Hospital, Shanxi, China; 6 Department of Nutrition and Food Safety, Hohhot Center for Disease Control and Prevention, Hohhot, Inner Mongolia China; 7 Department of Public Health, Xi’an Jiaotong University, Shaanxi, China; 8 Department of Social Medicine and Health Education, Peking University School of Public Health, Beijing, China; 9 School of Public Health, Faculty of Medicine, Imperial College London, London, UK; 10 UK Dementia Research Institute at Imperial College London, London, UK; 11 British Heart Foundation Centre for Research Excellence, Imperial College London, London, UK; 12 Department of Cardiology, Fuwai Hospital, Peking Union Medical College, Beijing, China; 13 Feinberg School of Medicine, Northwestern University, Chicago, IL USA

**Keywords:** Randomized trial, Sodium intake, Salt reduction, Blood pressure, Cardiovascular disease

## Abstract

**Background:**

Progressive reduction of sodium intake is an attractive approach for addressing excessive salt intake, but evidence for this strategy in real practice is limited. We aimed to determine the feasibility, effectiveness, and safety of a progressive sodium intake reduction intervention in real-world setting.

**Methods:**

We randomized 48 residential elderly care facilities in China, with 1612 participants aged 55 years and older, to either progressive reduction (PR, 24 facilities) or no reduction (NR, 24 facilities) of the supply of study salt to the kitchens of these facilities for 2 years. The primary efficacy outcome was systolic blood pressure (SBP) at any scheduled follow-up visit. Secondary efficacy outcomes included diastolic blood pressure (DBP) at any scheduled follow-up visit, and major adverse cardiovascular events (comprising non-fatal stroke, non-fatal myocardial infarction, hospitalized non-fatal heart failure, or vascular death) and total mortality. The perception of food saltiness, the addition of out-of-study salt in meals, and 24-h urinary sodium excretion were used as process indicators.

**Results:**

Pre-specified analysis per randomization found no effect of the intervention on the 2-year overall mean systolic and diastolic blood pressure (SBP, DBP) and any other outcomes. However, post hoc analysis showed that the intervention effect on blood pressure varied over multiple follow-up visits (*p* for interaction < 0.046) and presented favorable differences at the 24-month visit (SBP =  − 3.0 mmHg, 95%CI =  − 5.6, − 0.5; *p* = 0.020; DBP =  − 2.0 mmHg, 95%CI − 3.4, − 0.63; *p* = 0.004). The effect on 24-h sodium was non-significant (− 8.4 mmol, 95%CI =  − 21.8 to 4.9, *p* = 0.216), though fewer participants with NR than with PR reported food tasting bland (odds ratio 0.46; 95%CI 0.29 to 0.73; *p* = 0.001). Reporting of bland food taste and other process measures indicated that intervention delivery and adherence were not fully achieved as designed.

**Conclusions:**

The experience of this real-world study demonstrated that achieving acceptability and sustainability of the progressive sodium intake reduction strategy among older adults was challenging, but it has shown potential for effectiveness in these and potentially other residential settings if the lessons of DECIDE-Salt are applied in further studies.

**Trial registration:**

ClinicalTrials.gov (NCT03290716).

**Supplementary Information:**

The online version contains supplementary material available at 10.1186/s12916-023-03130-z.

## Background

High blood pressure is the leading cause of cardiovascular death globally [1], and the number of people with hypertension has doubled globally from 1990 to 2019 and reached over 1.25 billion in total [2]. Lowering dietary sodium intake reduces blood pressure and has been identified by the World Health Organization as one of the “best buys” to conquer the pandemic of chronic diseases [3, 4]. Scalable [5], practical, and effective strategies to lower dietary sodium intake require further development and evaluation.

Sodium consumption in China is among the highest in the world [6]. Progressive reduction in the use of salt for the preparation and seasoning of food is a recommended strategy for reducing dietary sodium consumption among elderly adults [7]. Small, stepwise reductions in the sodium content of foods could cumulate to significant decreases, while not being perceived by consumers [8]. Some small, short-term trials demonstrated that one-quarter decrease in the sodium content of bread can be delivered unnoticed with gradual reduction [9]. However, strong evidence for the effectiveness and feasibility of this strategy from large-scale, long-term studies remains lacking.

The DECIDE-Salt study aimed to determine the feasibility, effectiveness, and safety of two practical and scalable sodium reduction intervention strategies implemented in residential elderly care facilities: (1) replacing usual salt with salt substitute in facility kitchens and (2) making a progressive reduction (PR) in the quantity of study salt supplied to facility kitchens [10]. The main results of the trial have been published [11] and showed a mean 7.1 mmHg reduction in systolic and 1.9 mmHg reduction in diastolic blood pressure by replacing regular salt with salt substitute but not a significant reduction in blood pressure by the progressive reduction of study salt supply. The present paper presents the results of the post hoc analysis of the second intervention, with the aim to better understand these findings and learn experiences and lessons that could inform future studies.

## Methods

### Trial design and oversight

The DECIDE-salt study design has been published previously [10], and details of the main results were recently published [11]. Briefly, the study used a 2 × 2 factorial design, with 48 residential elderly care facilities in northern China being cluster-randomized to one of the four study groups: one with both interventions (PR and salt substitute), two with either intervention (NR and salt substitute; PR and usual salt), and one with no intervention (NR and usual salt). The baseline survey was initiated from September 2017 and the final follow-up was completed in October 2020. The study was approved by the Peking University Institutional Review Board and written informed consent was obtained from all participants. The trial was registered on ClinicalTrials.gov (NCT03290716).

### Participants

As a pragmatic trial, all residents in the facilities were put on the intervention for feasibility and practicality consideration, but the eligible population for the assessment of effectiveness was limited to those aged 55 years or older and had blood pressure measured at the baseline survey. The age limit was set to enhance the possibility to test the effect on the risk of major adverse cardiovascular event (MACE), the most important secondary outcome of the study, which increases with age.

### Randomization

Facilities were randomized in a 1:1:1:1 ratio to each of the four interventions using a central computerized process, with stratification by region. Random allocation of facilities was done after baseline survey data had been collected. The allocation codes are kept by the independent statistician.

### Intervention

The study salt (usual salt or salt substitute) supply to the kitchens in facilities allocated to the progressive reduction (PR) intervention group was reduced step by step, with the goal of achieving a facility-wide reduction of salt supply by 40% by the end of the intervention [10]. We recorded the study salt supply during the first month of intervention in each facility and the average amount per resident was computed to be the reference for setting the step-specific targets of the intervention in each facility. Responsible staff were trained to store the study salt supply in a locked room and supply it to the kitchen on a planned schedule, with a target of reduction by 5–10% every 3 months [10]. During each step, the cooks could use only the salt supplied; if additional salt was requested and delivered, that amount must be documented. At the end of each step, trained local investigators conducted a site visit to review the records on the amount of salt supply and storage. The mean salt intake level per person was calculated at the end of each step to assess whether the planned stepwise target had been achieved. Once the facility successfully achieved the planned target, the next step of salt reduction target was initiated. Otherwise, the previous planned target was retained for the next step, and the cook and responsible staff for salt supply control were retrained. During the intervention, table salt (study salt) was allowed to help individuals who had difficulty adjusting to the change. At the intervention kick-off event, a health education lecture on the importance of salt reduction and the plan for the program was given to all residents. Posters were also used to encourage the residents to support the sodium reduction program. At every monitoring visit, the study staff responsible for the PR intervention reinforced the implementation messages to aid the planned targets being achieved.

The study salt supply to the kitchens in facilities allocated to the usual supply group was not restricted. The study salt, either salt substitute or usual salt, was provided centrally every 3 months and for free.

### Follow-up and outcomes

Follow-up was scheduled for 6, 12, 18, and 24 months after randomization. The outcome assessment team was independent of the staff responsible for implementing the interventions. The primary efficacy outcome SBP at any scheduled follow-up visit during 24 months (except for participants in Xi’an where follow-up was conducted at 12 and 24 months, due to lack of personnel). Three measurements of blood pressure were recorded using an OMRON HEM-7136 device following American Heart Association guidelines [12]. Secondary efficacy outcomes included DBP at any scheduled follow-up visit during 24 months, major adverse cardiovascular events adjudicated as definite (comprising non-fatal stroke, non-fatal myocardial infarction, hospitalized non-fatal heart failure or vascular death), and total mortality in 2 years of intervention. Indicators of salt supply reduction included 24-h urinary sodium and perception of food saltiness. Safety outcomes included hyponatremia (defined as serum sodium < 135mmol/L) and serum sodium level. Both serum and urinary sodium were measured at both baseline and 24 months, with the ion-selective electrode method [13] on a Roche Cobas c501 platform. The perception of food saltiness at the baseline and every follow-up visit was measured by the closed question ‘how do you feel the food that is provided in your kitchen?’ with the optional answers: bland, okay, and salty. We also questioned the participants at the 24-month visit if they added out-of-study salt to their meals when they felt the food was bland.

### Sample size and statistical analysis

The study was designed to provide 80% statistical power (with two-sided alpha = 0.05) to detect a minimum difference of 3.0mmHg SBP between randomized groups [10].

#### Analyses on effect on blood pressure

The primary analysis on the effect on blood pressure was assessed among participants who had at least one blood pressure measurement during follow-up (1219 participants). The model for analysis pre-specified in the statistical analysis plan (SAP) was a linear mixed model [14] for repeat measures, accounting for clustering at the facility level, and assuming that the intervention effect was constant over 2 years. That assumption did not reflect the nature of the progressive intervention; hence, we changed the model to allow the effect of intervention to vary by the follow-up visit in the present post hoc analysis. Results from both models are given. Details of the model used are provided in supplementary Additional file 2.

Secondary analyses on the intervention effect on blood pressure were done with per-protocol analysis (1195 participants); multiple imputation for missing follow-up values (1612 participants); and adjustment for age, sex, and region (1219 participants), with exclusion of data from facilities in Xi’an (1019 participants), according to the pre-defined statistical analysis plan.

Possible variation in intervention effect by season was explored using a linear mixed model that allows the effect of intervention to vary by season (see Additional file 2). According to the climate in northern China, we defined the warm season to include the time span from April 1 to September 30 and the rest of the year as the cold season.

#### Analyses on effect on cardiovascular events and mortality

The analyses for cardiovascular events and mortality were based on the first occurrence of each event among the 1612 participants. And the results have been reported previously [11].

#### Analyses on effect on 24-h urinary sodium excretion

The same model as that pre-specified in the SAP for the primary outcome was used to calculate the effects on 24-h urinary sodium in the 639 participants with measurements at both baseline and 24 months.

#### Analyses on effect on serum sodium and hyponatremia

The same model as for the primary outcome in post hoc analysis was used to calculate the effects on serum sodium. Assessment of hyponatremia was done by estimation from generalized linear mixed models with adjustment for clustering [15].

#### Analyses on effect on perception of food saltiness and behavior of self-addition of out-of-study salt

We compared the participants’ perception of food saltiness between randomized groups across the four follow-up visits during 2 years of intervention and further analyzed the association of participants’ reporting food as bland with self-addition of out-of-study salt, using a generalized linear mixed model among all participants adjusting for the intervention.

In addition, we did sensitivity analysis separately in participants who reported and did not report the out-of-study salt addition using similar statistical models as aforementioned.

All analyses were done using SAS version 9.4. The intervention effect was summarized as point estimates and 95% confidence intervals (CI) with *p*-values. We did not adjust for multiple tests in our analyses.

## Results

### Study implementation and baseline characteristics

Among 48 residential elderly care facilities, 1612 individuals aged 55 years or older had their blood pressure measured at baseline and thus were eligible for the study. These eligible participants had a mean age of 71.0 years and 76.3% were men. Other baseline characteristics are shown in Table 1 and all were balanced across randomized groups.

**Table 1:** Baseline characteristics of study participants by randomized groups (*n* = 1612)

	PR (*n* = 843)	NR (*n* = 769)
**Cluster level**		
Number of facilities, *n*	24	24
Number of study participants per facility, median (IQR)	30 (20,44)	28 (23,39)
**Individual level**		
**Demographics and anthropometrics**		
Age, years, mean ± SD	71.6 ± 9.8	70.5 ± 9.2
Male, *n* (%)	623 (73.9)	607 (78.9)
Study site, *n* (%)		
Changzhi	279 (33.1)	210 (27.3)
Xi’an	228 (27.1)	267 (34.7)
Hohhot	184 (21.8)	150 (19.5)
Yangcheng	152 (18.0)	142 (18.5)
Education at junior high school or above, *n* (%)	270 (34.9)	215 (29.7)
** Life style**		
Current smokers, *n* (%)	277 (32.9)	262 (34.1)
Current alcohol drinker, *n* (%)	79 (9.4)	77 (10.0)
** Unhealthy status**		
Hypertension, *n* (%)	522 (61.9)	479 (62.3)
Coronary artery disease, *n* (%)	79 (9.4)	67 (8.7)
Stroke, *n* (%)	181 (21.5)	201 (26.1)
Diabetes, *n* (%)	84 (10.0)	80 (10.4)
Renal disease, *n* (%)	49 (5.8)	39 (5.1)
Bedridden or other severe disease, *n* (%)	49 (5.8)	54 (7.0)
Any of above	611 (72.5)	578 (75.2)
** Medication use**		
Anti-HTN Med, *n* (%)	337 (40.0)	286 (37.3)
** Blood pressure and lab measures**		
SBP, mmHg, mean ± SD	137.1 ± 20.9	138.0 ± 21.8
DBP, mmHg, mean ± SD	79.9 ± 11.2	81.2 ± 12.0
Serum sodium, mmol/L, mean ± SD	142.7 ± 2.5	142.4 ± 2.4
24-h urinary sodium, mmol, mean ± SD	164.2 ± 83.9	161.9 ± 81

There were some cases with missing data at the baseline for anti-hypertensive medication (*n* = 3) and urinary sodium and potassium (*n* = 484)

*PR* progressive reduction, *NR* no reduction

**Fig. 1:**
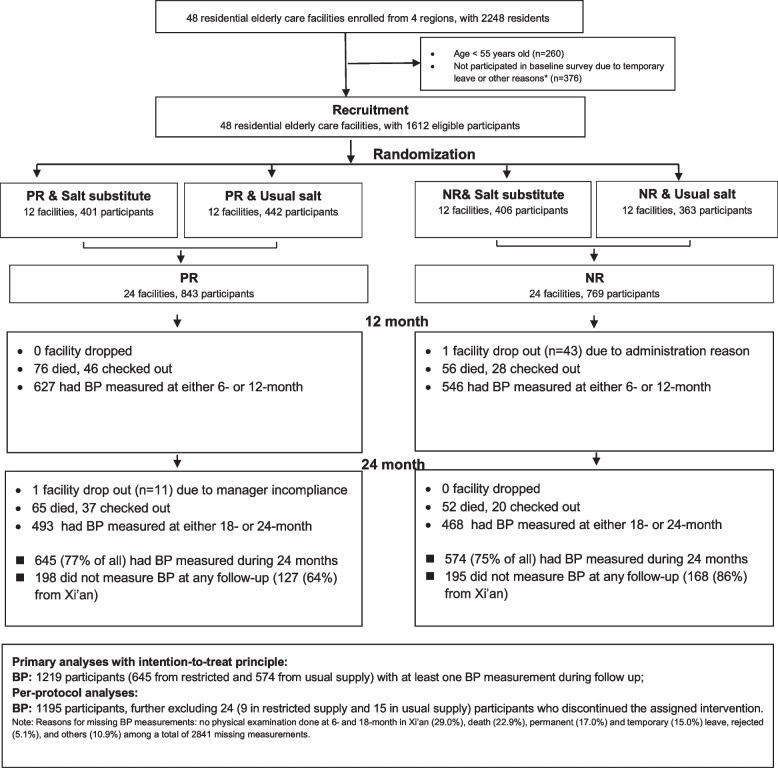
Flowchart. Note: Follow-up at 6 and 18 months were only for blood pressure (BP) measurement and not shown in Fig. 1, and 903 and 799 were measured respectively. *Reasons for not participating in the baseline survey: 185 (49.2%) were on temporary leave (mostly for treatment of their illness at hospitals, some for personal business such as travel to the relatives), 33 (8.8%) were handicapped persons (with deaf, mute, blindness, dementia, etc.), 59 (15.7%) were with severely ill or bedridden, 32 (8.5%) refused baseline survey, and 67 (17.8%) with unknown reasons

Among all 1612 eligible participants, 1219 (76%) had at least one follow-up blood pressure measured, 1193 (74%) had data on the perception of food taste and behavior of personal addition of out-of-study salt, and 1086 participants (67%) had blood samples tested at baseline and either 12 or 24 months, but only 639 (40%) had 24-h urine collected (Fig. 1). The percentages of participants eligible for the analysis were similar between intervention groups for all study outcomes (Additional file 1: Table S1-1). For each study outcome (blood pressure, urinary sodium excretion, and reported food taste), compared with those not included in the analysis, the group included in the analysis were younger, fewer women, more smokers and alcohol users, less educated, more with hypertension and on anti-hypertension medication, fewer bedridden, or severely ill (Additional file 1: Table S1-2, S1-3, and S1-4). However, none of the baseline characteristics differed between intervention groups (Additional file 1: Table S1-2, S1-3 and S1-4).

### Effects on blood pressure

**Fig. 2:**
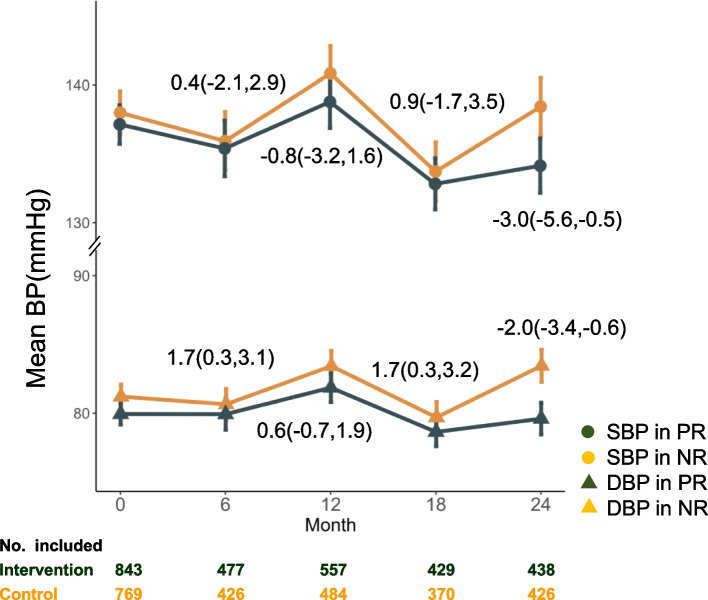
Effects on systolic and diastolic blood pressure of PR versus NR during the 2 years of intervention in DECIDE-salt study. The figure shows the mean and 95%CI of SBP (upper) and DBP (lower) at baseline and each follow-up visit for participants who had their blood pressure measured at each visit. The numbers beside the BP point were mean differences and 95%CI in SBP and DBP at each follow-up visit between comparison groups, which were estimated using a linear mixed model with repeated measurements, accounting for the clustering effect and adjusting for the time of visits as well as time-by-group interaction, among 1219 participants

The pre-specified analysis showed that the overall mean SBP during 2 years was not significantly reduced, nor DBP. However, the post hoc analysis revealed a temporal and seasonal variation in the intervention effect. At the 24-month visit, PR compared to NR showed a reduction in both SBP (− 3.0 mmHg; 95%CI =  − 5.6 to − 0.5 mmHg, *p* = 0.020, *p* for interaction = 0.046) and DBP (− 2.0 mmHg; 95%CI =  − 3.4, − 0.6, *p* = 0.005, *p* for interaction < 0.001) (Fig. 2). The intervention effect also differed between warm and cold seasons (*p* for interaction < 0.001 for both SBP and DBP), though neither estimate individually was statistically significant (Table 2).

**Table 2:** Changes in systolic and diastolic blood pressure in PR versus NR, in 1219 participants and participants by addition of extra salt from outside of study

Variables	PR	NR	All participants (*n* = 1219)			Not adding extra salt (*n* = 716)		Adding extra salt (*n* = 95)	
			**Difference**	***p* value**	***p* for interaction**	**Difference**	***p* for interaction**	**Difference**	***p* for interaction**
	***n* = 645**	***n* = 574**							
**Systolic blood pressure**									
Changes from baseline by visit									
6-month	− 3.1 ± 20.8	− 3.1 ± 19.6	0.4 (− 2.1, 2.9)	0.741	0.046	0.2 (− 2.9, 3.3)	0.076	7.2 (− 1.4, 15.8)	0.412
12-month	0.8 ± 21.1	1 ± 20.3	− 0.80 (− 3.2, 1.6)	0.510		− 1.3 (− 4.2, 1.7)		1.8 (− 6.6, 10.2)	
18-month	− 5.1 ± 20.5	− 5.9 ± 19.8	0.9 (− 1.7, 3.5)	0.509		− 0.2 (− 3.3, 2.9)		6.6 (− 2.2, 15.4)	
24-month	− 4 ± 20.5	− 0.7 ± 20.6	− 3.0 (− 5.6, − 0.5)	0.020		− 3.6 (− 6.5, − 0.7)		2.2 (− 6.1, 10.5)	
Changes from baseline by season									
Warm season	− 4.4 ± 18.1	− 6.9 ± 18.2	2.8 (− 1.4, 6.9)	0.191	< 0.001	2.5 (− 2.0, 6.9)	< 0.001	7.3 (− 1.4, 16.0)	0.063
Cold season	− 1.1 ± 19.9	0.5 ± 19	− 2.1 (− 6.1, 1.9)	0.300		− 2.3 (− 6.5, 1.9)		0.9 (− 7.0, 8.8)	
Mean change over 2 years									
Overall change^a^	− 2.5 ± 18.6	− 2.1 ± 18.1	− 0.6 (− 4.5, − 3.4)	0.774		− 0.8 (− 5.0, 3.4)		3.3 (− 4.0, 10.7)	
**Diastolic blood pressure**									
Changes from baseline by visit									
6-month	− 0.8 ± 11.4	− 2.5 ± 10.2	1.7 (0.3, 3.1)	0.018	< 0.001	2.0 (0.2, 3.7)	< 0.001	4.6 (− 0.2, 9.2)	0.071
12-month	1.5 ± 11.3	0.5 ± 10.6	0.6 (− 0.7, 1.9)	0.371		0.2 (− 1.5, 1.8)		3.1 (− 1.4, 7.6)	
18-month	− 2.0 ± 10.9	− 4.0 ± 10.2	1.7 (0.3, 3.2)	0.021		1.1 (− 0.6, 2.8)		4.8 (0.0, 9.5)	
24-month	− 1.3 ± 11.0	0.8 ± 11.3	− 2.0 (− 3.4, − 0.6)	0.0045		− 2.3 (− 3.9, − 0.6)		− 0.4 (− 4.9, 4.0)	
Changes from baseline by season									
Warm season	− 1.1 ± 9.9	− 3.9 ± 8.6	2.4 (0.4, 4.3)	0.017	< 0.001	2.3 (0.1, 4.5)	< 0.001	3.6 (− 0.7, 8.0)	0.022
Cold season	0.3 ± 10.5	0.7 ± 9.9	− 0.9 (− 2.7, 1.0)	0.351		− 1.1 (− 3.1, 0.9)		− 0.7 (− 4.6, 3.3)	
Mean change over 2 years									
Overall change^a^	− 0.4 ± 9.8	− 0.9 ± 9.5	0.2 (− 1.6, 1.9)	0.8461		− 0.0 (− 2.0, 1.9)		0.9 (− 2.8, 4.6)	

^a^Information on the addition of out-of-study salt was collected only at 24-month follow-up and was missing for 408 participants. The analysis included only participants with the information available

*PR* progressive reduction, *NR* no reduction

Secondary analyses showed similar results as that shown in our primary analysis (Additional file 1: Table S2-1, Table S2-2, Table S2-3 and Table S2-4).

### Effects on process indicators

**Fig. 3:**
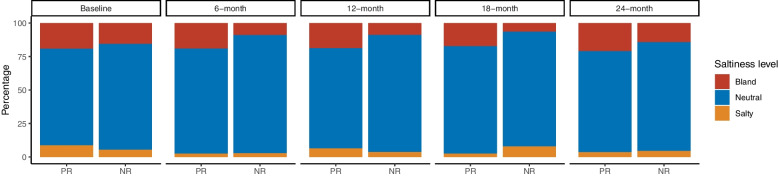
Self-reported feel of food saltiness among study participants with PR versus NR. Among participants who completed the questionnaire at baseline and at least one follow-up visit (*N* = 1193), participants with NR reporting the meals tasted bland were significantly less compared to those with PR (odds ratio 0.46; 95%CI 0.29 to 0.73; *p* = 0.001) during 2 years of intervention

Compared with PR, fewer participants with NR stated that the meals were bland during the whole intervention period (odds ratio 0.46; 95%CI 0.29 to 0.73; *p* = 0.001) (Fig. 3) although 15% of participants in facilities with NR reported food was bland at baseline too, which was similar to that for those in PR (15% vs. 17%, *p* = 0.342).

Further analysis showed that those who reported food as bland tended to be more likely to add out-of-study salt in their meals (Additional file 1: Table S3). Analysis excluding participants who reported self-addition of out-of-study salt confirmed the effect on blood pressure but with larger point estimations (Table 2).

Mean 24-h urinary sodium excretion was not significantly reduced (− 8.4 mmol; 95%CI =  − 21.8 to 4.9 mmHg, *p* = 0.216) in participants with PR compared to that with NR.

### Effects on safety outcomes

There was no effect on serum sodium (difference 0.01; 95%CI =  − 0.58 to 0.61, *p* = 0.968) or risk of hyponatremia (RR 1.04; 95%CI = 0.33 to 3.21, *p* = 0.952) in participants with PR compared to those with NR.

### Case studies

Case study results showed that the intervention effects on different outcomes were generally internally coherent within each facility but varied from facility to facility and the variation did not necessarily reflect the intervention assignment. Four typical cases with successful and unsuccessful interventions are given in the Additional file 1: Table S4.

## Discussion

Our post hoc statistical model that allows for the variation in effect by the time of intervention, which may fit better the nature of the intervention, showed a favorable effect of the progressive reduction of salt supply on both SBP and DBP at the end of 2 years of intervention, although this was not seen in the analysis per protocol. While 24-h urinary sodium excretion from a small subset of study participants was not reduced in the intervention arm, the effects on self-reported food tasting and self-addition of out-of-study table salt in a larger subset, as well as the results from the analysis excluding those who reported self-addition of out-of-study table salt, suggest that sodium intake in the PR group may have been reduced and may have contributed to the observed blood pressure differences. However, these results differed from the pre-specified model that respected the randomization but assumed an intervention effect that did not vary over time [11]. It is therefore important to outline some aspects of the incremental sodium reduction implementation in DECIDE-Salt to help us understand the intervention.

The premise of the strategy of gradual incremental reduction of dietary salt is that this policy could be introduced unobtrusively for a population, or a group. The sodium content of foods could then be reduced in a progressive step-wise manner, allowing adaptation of consumers’ taste before each step [16, 17]. This was the intent of the salt reduction intervention in this trial.

However, in the particular circumstances of DECIDE-Salt, from early in the trial self-reports of food tasting bland as well as action of some participants to add out-of-study table salt, both occurred more frequently in the intervention than in the control arm—supporting two important conclusions: First, salt reduction appeared to be achieved by the intervention but, second, the intent to introduce salt reduction unobtrusively in the whole diet was not fully successful. The strategy applied in prior studies was generally limited to a single food such as bread or only a few selected foods, perhaps making the reduction of salt content less likely to be recognized [8, 9, 17]. To our knowledge, the DECIDE-Salt trial was the first to try the stepwise approach to lower salt use in the whole diet.

Similar, though less frequent, self-reports of blandness of the food were received from participants in the control arm. It is possible that some of the control facilities may have adopted a salt reduction policy on their own, in disregard of the protocol. Our findings from the case studies, which integrated the information on self-reported blandness of food, urinary sodium, and blood pressure, indicated that salt reduction may have taken place in some control facilities. The high proportion of participants reporting food blandness at baseline in both intervention and control groups also supports this idea. Although this did not affect the balance of intervention groups because the randomization was done after baseline, it may have reduced the potential impact of the salt reduction intervention, leaving less room for the intervention to take effect. For future studies, to prevent such “spontaneous intervention” before the actual intervention, the kick-off meeting and training of the facilities’ managers should emphasize that they should strictly follow the study protocol to implement the study interventions assigned to them.

A factor limiting the evaluation of the salt reduction intervention was the health status of the study participants. Co-morbidity frequently limited the completeness of follow-up. The reduced effective sample size and observed numbers of detectable events may have limited the statistical power of the study to identify potential differences in outcomes, e.g., 24-h urinary sodium, for which only 639 participants had data at 24 months for analysis.

Despite these difficulties, the study achieved over 75% of participants with follow-up blood pressure measured for the assessment of effects on primary outcome, and the characteristics of those participating were similar between the study arms, as noted above.

The difficulty we had in reducing sodium intake and blood pressure through the progressive reduction of salt supply demonstrated that it is not easily achieved and may help to explain why the WHO global target for population sodium reduction by 30% by 2025 would not be achieved [18] if no effective strategies other than mass health education is developed and rolled out globally.

Long-term reduction of salt intake requires effort to achieve the change in dietary behavior of the consumers unless the change cannot be detected, the change is within the tolerable range, or the consumers are prepared and ready for the change. In the DECIDE-Salt trial, only a simple health education program was given, and the participants may have been poorly prepared for the intervention. In such circumstances, the detection of salt reduction in foods by the consumers through the accompanying change in food taste might lead to their opposition to the intervention [19, 20]. We would suggest to add in a health education program before initiating the salt supply reduction program so that the facilities’ residents are well prepared for and may more readily accept the intervention, and briefly but continuously reinforce the messaging during the implementation of the salt supply reduction program to help to maintain enthusiasm for the intervention.

Further insights into the strategy of unobtrusive salt reduction are important for the design and implementation of future studies. (1) Where cooperation of an institutional gatekeeper such as kitchen management is required, thorough orientation to the study is needed for those involved in both arms of the trial—its rationale, potential benefits, and need to adhere strictly to the protocol to permit evaluation. (2) Persistence of study oversight and monitoring of fidelity to the intervention design are necessary over the full course of the trial. (3) To permit ongoing assessment of changes in salt intake, a subsample of willing participants should provide 24-h urine collections at each step for analysis of sodium excretion. (4) An interesting question for further study is whether seasonal variation in climate is associated with customary changes in salt intake or salt taste [21]. Due to Chinese culture and the variability in the availability of fresh foods between seasons, people in northern China like to have low salt, low-fat dishes with more fresh vegetables and fruits in warm seasons and warm and meaty dishes that require more salt added to satisfy the same taste in cold seasons; for trial settings with collective catering, the answer may be to suggest special measures over the course of study, e.g., different diets for summer and winter seasons. Also important is the assurance of comparability between intervention groups in the timing of evaluations with respect to the season. (5) Composition of a candidate study population should be considered with respect to age, sex, comorbidities, and other factors in order to optimize compliance [22], outcome assessment, and generalization to other populations. (6) Consideration should be given to extending post-intervention follow-up to assess the sustainability of the intervention and its benefits long-term. An alternative, and potentially complementary strategy, is the provision of sodium-reduced, potassium-enhanced table salt for use in cooking and at table, which has been shown in the SSaSS trial [23] in China to reduce both blood pressure and cardiovascular events.

## Conclusions

To conclude, the DECIDE-Salt efforts to progressively reduce the supply of study salt to facility kitchens and by this means to reduce the sodium intake of study participants met with mixed success after 2 years of intervention. Lessons to improve adherence to sodium reduction strategies learned from DECIDE-Salt may be helpful in the design of future studies.

### Supplementary Information

**Additional file 1: Table S1.** Evaluation of possible bias from missing data in main results in DECIDE-Salt trial. **Table** **S1-1.** Comparison between intervention groups in percentages of participants actually analyzed for the assessment of effect on blood pressure and 24-hr urine electrolytes. **Table** **S1-2.** Comparisons between participants analyzed and not analyzed in baseline characteristics, among 1612 eligible participants for the assessment of effect on blood pressure. **Table** **S1-3.** Comparisons between participants analyzed and not analyzed in baseline characteristics, among 1612 eligible participants for the assessment of effect on urinary sodium. **Table** **S1-4.** Comparisons between participants analyzed and not analyzed in baseline characteristics, among 1612 eligible participants for the assessment of food saltiness. **Table** **S2.** Secondary analysis on effect on blood pressure. **Table S2-1.** Changes in systolic and diastolic blood pressure in PR versus NR of study salt, in participants excluding Xi’an (*N* = 1019) and participants by addition of extra salt from outside of study. **Table S2-2.** Changes in systolic and diastolic blood pressure in PR versus NR of study salt, in 1195 participants (Per protocol analysis) and participants by addition of extra salt from outside of study in per-protocol analysis. **Table** **S2-3.** Age, sex- and center-adjusted changes in systolic and diastolic blood pressure in PR versus NR of study salt, in 1219 participants and participants by addition of extra salt from outside of study, adjustment for age, sex and region. **Table** **S2-4.** Changes in systolic and diastolic blood pressure in PR versus NR of study salt in analysis with imputed follow-up measurements. **Table** **S3.** Association of participants’ reporting on food taste with personal-addition of out-of-study salt at 24-month follow up. **Table** **S4.** Examples of variation between facilities on BP, urinary sodium, saltiness and salt addition.

**Additional file 2.** Supplementary Appendix on statistical methods.

## Data Availability

Data will be disclosed only on request and approval of the proposal by the study review committee. Deidentified participant data and a data dictionary will be made available following approval. A detailed research protocol and statistical analysis plan will be shared as supplements to this publication.

## Abbreviations

Anti-HTN Med: Anti-hypertensive medication
CI: Confidence Interval
DBP: Diastolic blood pressure
DECIDE-Salt: Diet, ExerCIse and carDiovascular hEalth—Salt Reduction Strategies for the Elderly in Nursing Homes in China
MACE: Major adverse cardiovascular events
NR: No restriction
PR: Progressive reduction
SAP: Statistical analysis plan
SBP: Systolic blood pressure
