# Supplementary material for: Experience with 2 years’ intervention to progressively reduce salt supply to kitchens in elderly care facilities—challenges and further research: post hoc analysis of the DECIDE-Salt randomized clinical trial
Source: BMC Med. 2023 Nov 3;21:416. doi: 10.1186/s12916-023-03130-z (PMC10623877; doi:10.1186/s12916-023-03130-z)

**Table S1.** **Evaluation of possible bias from missing data in main results in DECIDE-Salt trial**

Table S1-1. Comparison between intervention groups in percentages of participants actually analyzed for the assessment of effect on blood pressure and 24-hr urine electrolytes

|  | | Total | PR | NR | p |
| --- | --- | --- | --- | --- | --- |
| All residents | No. | 1612 | 843 | 769 |  |
| Participants actually analyzed for primary outcome assessment | No. | 1219 | 645 | 574 |  |
|  | % in eligible participants | 75.6 | 76.5 | 74.6 | 0.3824 |
| Participants actually analyzed for 24-hr urine electrolytes | No. | 639 | 342 | 297 |  |
|  | % in eligible participants | 39.6 | 40.6 | 38.6 | 0.4246 |
| Participants actually analyzed for food saltiness | No. | 1193 | 629 | 564 |  |
|  | % in eligible participants | 74.0 | 74.6 | 73.3 | 0.5607 |

Table S1-2. Comparisons between participants analyzed and not analyzed in baseline characteristics, among 1612 eligible participants for the assessment of effect on blood pressure

|  | Analyzed (n=1219) | | |  | Not analyzed (n=393) | | | P# |
| --- | --- | --- | --- | --- | --- | --- | --- | --- |
|  | PR(N=645) | NR(N=574) | p |  | PR(N=198) | NR (N=195) | p |  |
| % of women | 137 (21.2%) | 65 (11.3%) | 0.9996 |  | 83 (41.9%) | 97 (49.7%) | 0.9997 | 0.0177 |
| Mean age | 70.6± 9.4 | 68.6± 8.0 | 0.6308 |  | 74.6± 10.2 | 75.9± 10.1 | 0.8251 | 0.0007 |
| junior high or above | 189 (30.8%) | 133 (23.6%) | 0.1293 |  | 81 (50.6%) | 82 (50.6%) | 0.3305 | 0.0209 |
| % of current smoker | 246 (39.0%) | 242 (43.0%) | 0.6027 |  | 31 (18.8%) | 20 (12.4%) | 0.5315 | 0.0016 |
| % of current drinker | 67 (10.6%) | 68 (12.1%) | 0.493 |  | 12 (7.3%) | 9 (5.6%) | 0.5189 | 0.0373 |
| Mean BMI | 24.2± 3.4 | 24.0± 3.5 | 0.6196 |  | 23.4± 3.5 | 24.9± 3.9 | 0.0442 | 0.7740 |
| % of HTN | 423 (65.6%) | 377 (65.7%) | 0.9772 |  | 99 (50.0%) | 102 (52.3%) | 0.3823 | <0.0001 |
| % of CVD | 180 (34.4%) | 156 (32.6%) | 0.5266 |  | 54 (40.9%) | 79 (51.3%) | 0.6409 | 0.2126 |
| % of Bedridden or other severe disease | 28 (5.4%) | 17 (3.6%) | 0.3273 |  | 21 (16.0%) | 37 (24.2%) | 0.5381 | 0.0005 |
| % of anti-HTN med. | 278 (43.1%) | 219 (38.2%) | 0.1888 |  | 59 (30.0%) | 67 (34.5%) | 0.1595 | 0.0044 |
| SBP | 138.2± 21.3 | 139.1± 21.3 | 0.6543 |  | 133.8± 19.2 | 134.7± 23 | 0.1787 | 0.0566 |
| DBP | 80.5± 11.6 | 82.3± 11.81 | 0.5416 |  | 78.1± 9.5 | 78.0± 12.2 | 0.3885 | 0.1960 |

*p value was obtained from models to compare baseline characteristics between participants assigned with PR versus NR among analysed and not analysed, accounting for clustering at facility level.

#p value was obtained from models to compare baseline characteristics between participants analysed and not analysed, accounting for clustering at facility level

Table S1-3. Comparisons between participants analyzed and not analyzed in baseline characteristics, among 1612 eligible participants for the assessment of effect on urinary sodium

|  | Analyzed (n=639) | | |  | Not analyzed (n=973) | | | P# |
| --- | --- | --- | --- | --- | --- | --- | --- | --- |
|  | PR (N=342) | NR (N=297) | p |  | PR (N=501) | NR (N=472) | p |  |
| % of women | 47 (13.7%) | 18 (6.1%) | 0.1928 |  | 173 (34.5%) | 144 (30.5%) | 0.1928 | <.0001 |
| Mean age | 68.5± 8.3 | 67.0± 6.7 | 0.1117 |  | 73.6± 10.1 | 72.7± 9.8 | 0.7094 | <.0001 |
| junior high or above | 98 (29.1%) | 75 (25.4%) | 0.2473 |  | 172 (39.5%) | 140 (32.6%) | 0.1002 | 0.199 |
| % of current smoker | 161 (47.6%) | 134 (45.6%) | 0.9193 |  | 116 (25.3%) | 128 (29.7%) | 0.7868 | 0.0088 |
| % of current drinker | 47 (13.9%) | 40 (13.6%) | 0.9064 |  | 32 (7.0%) | 37 (8.6%) | 0.4631 | 0.0016 |
| Mean BMI | 24.2± 3.3 | 24.1± 3.3 | 0.884 |  | 24.0± 3.6 | 24.2± 3.9 | 0.891 | 0.5539 |
| % of HTN | 220 (64.3%) | 196 (66.0%) | 0.867 |  | 302 (60.3%) | 283 (60.0%) | 0.912 | 0.363 |
| % of CVD | 105 (33.3%) | 95 (35.2%) | 0.9987 |  | 129 (37.8%) | 140 (38.7%) | 0.4455 | 0.6514 |
| % of Bedridden or other severe disease | 10 (3.2%) | 4 (1.5%) | 0.2085 |  | 39 (11.7%) | 50 (14.0%) | 0.918 | 0.0001 |
| % of anti-HTN med. | 145 (42.4%) | 120 (40.4%) | 0.445 |  | 192 (38.4%) | 166 (35.3%) | 0.5425 | 0.2147 |
| SBP | 137.9± 19.9 | 138.8± 21.2 | 0.6668 |  | 136.6± 21.5 | 137.5± 22.1 | 0.9539 | 0.77 |
| DBP | 81.0± 10.6 | 82.7± 11.4 | 0.1928 |  | 79.2± 11.6 | 80.3± 12.3 | 0.9356 | 0.4608 |

*p value was obtained from models to compare baseline characteristics between participants assigned with PR versus NR among analysed and not analysed, accounting for clustering at facility level.

#p value was obtained from models to compare baseline characteristics between participants analysed and not analysed, accounting for clustering at facility level

Table S1-4. Comparisons between participants analyzed and not analyzed in baseline characteristics, among 1612 eligible participants for the assessment of food saltiness

|  | Analyzed (n=1193) | | |  | Not analyzed (n=419) | | | P# |
| --- | --- | --- | --- | --- | --- | --- | --- | --- |
|  | PR (N=629) | NR (N=564) | p |  | PR (N=214) | NR (N=205) | p |  |
| % of women | 130 (20.7%) | 57 (10.1%) | 0.9996 |  | 90 (42.1%) | 105 (51.2%) | 0.6006 | 0.3718 |
| Mean age | 70.6± 9.4 | 68.4± 7.9 | 0.4889 |  | 74.5± 10.2 | 76.2± 10.0 | 0.9855 | <.0001 |
| junior high or above | 180 (30.0%) | 132 (23.7%) | 0.1775 |  | 90 (52.0%) | 83 (49.1%) | 0.2583 | 0.0247 |
| % of current smoker | 245 (39.6%) | 242 (43.6%) | 0.6129 |  | 32 (18.0%) | 20 (11.8%) | 0.5418 | 0.0002 |
| % of current drinker | 67 (10.8%) | 68 (12.3%) | 0.518 |  | 12 (6.8%) | 9 (5.3%) | 0.5632 | 0.0127 |
| Mean BMI | 24.2± 3.4 | 24.0± 3.5 | 0.6196 |  | 23.4± 3.5 | 24.9± 3.9 | 0.0442 | 0.774 |
| % of HTN | 415 (66.0%) | 371 (65.8%) | 0.9062 |  | 107 (50%) | 108 (52.7%) | 0.3594 | <0.0001 |
| % of CVD | 180 (34.4%) | 156 (32.6%) | 0.5266 |  | 54 (40.9%) | 79 (51.3%) | 0.6409 | 0.2126 |
| % of Bedridden or other severe disease | 28 (5.4%) | 17 (3.6%) | 0.3273 |  | 21 (16.0%) | 37 (24.2%) | 0.5381 | 0.0005 |
| % of anti-HTN med. | 272 (43.2%) | 216 (38.3%) | 0.1899 |  | 65 (30.5%) | 70 (34.5%) | 0.2113 | 0.003 |
| SBP | 138.3± 21.4 | 139.1± 21.2 | 0.7552 |  | 133.7± 18.8 | 135.0± 23.2 | 0.1561 | 0.0599 |
| DBP | 80.6± 11.7 | 82.4± 11.8 | 0.6006 |  | 78.1± 9.3 | 78.1± 12.3 | 0.3718 | 0.2205 |

*p value was obtained from models to compare baseline characteristics between participants assigned with PR versus NR among analysed and not analysed, accounting for clustering at facility level.

#p value was obtained from models to compare baseline characteristics between participants analysed and not analysed, accounting for clustering at facility level

**Table S2 Secondary analysis on effect on blood pressure**

# **Table S2-1 Changes in systolic and diastolic blood pressure in PR versus NR of study salt, in participants excluding Xi’an (N = 1019) and participants by addition of extra salt from outside of study**

| **Variables** |  | **PR** | **NR** | **All participants(n=1019)** | | |  | **Not adding extra salt(n=936)** | |  | **Adding extra salt(n=83)** | |
| --- | --- | --- | --- | --- | --- | --- | --- | --- | --- | --- | --- | --- |
|  |  |  |  | **Difference** | **p value** | **p for interaction** |  | **Difference** | **p for interaction** |  | **Difference** | **p for interaction** |
|  |  | **n = 544** | **n = 475** |  |  |  |  |  |  |  |  |  |
| **Systolic blood pressure** | | | | | | | | | | | | |
| Changes from baseline by visit* | | | | | | | | | | | | |
| 6-month |  | -3.1±20.8 | -3.1±19.6 | 0.27(-2.25,2.8) | 0.8322 | 0.068 |  | -0.4(-3.04,2.25) | 0.069 |  | 7.25(-1.35,15.85) | 0.338 |
| 12-month |  | 0.2±20.3 | 1.2±19.5 | -1.34(-3.91,1.23) | 0.3083 |  |  | -1.45(-4.14,1.23) |  |  | 0.44(-8.32,9.2) |  |
| 18-month |  | -5.1±20.5 | -5.9±19.8 | 0.74(-1.9,3.38) | 0.583 |  |  | 0.15(-2.62,2.91) |  |  | 6.58(-2.21,15.37) |  |
| 24-month |  | -3.9±20.1 | -1.3±20.6 | -2.89(-5.53,-0.24) | 0.0326 |  |  | -3.59(-6.38,-0.81) |  |  | 3.71(-4.8,12.21) |  |
| Changes from baseline by season^&^ | | | | | | | | | | | | |
| warm season |  | -4.3±20.3 | -6.7±19.5 | 2.45(-1.8,6.69) | 0.2582 | < 0.001 |  | 2.11(-2.2,6.43) | <0.001 |  | 7.41(-0.59,15.42) | 0.061 |
| cold season |  | -2±20.6 | 0.9±19.8 | -2.41(-6.55,1.73) | 0.253 |  |  | -2.66(-6.86,1.54) |  |  | 0.96(-6.49,8.42) |  |
| **Diastolic blood pressure** | | | | | | | | | | | | |
| Changes from baseline by visit* | | | | | | | | | | | | |
| 6-month |  | -0.8±11.4 | -2.5±10.2 | 1.77(0.38,3.16) | 0.0127 | <0.001 |  | 1.5(0.04,2.95) | <0.001 |  | 4.58(-0.11,9.28) | 0.127 |
| 12-month |  | 1.3±10.9 | 0.3±9.8 | 0.7(-0.71,2.11) | 0.3319 |  |  | 0.49(-0.99,1.97) |  |  | 3.09(-1.68,7.87) |  |
| 18-month |  | -2±10.9 | -4±10.2 | 1.81(0.36,3.26) | 0.0146 |  |  | 1.51(-0.01,3.04) |  |  | 4.77(-0.02,9.56) |  |
| 24-month |  | -1.4±10.6 | 0.3±11.1 | -1.93(-3.39,-0.48) | 0.0092 |  |  | -2.16(-3.7,-0.63) |  |  | 0.28(-4.36,4.92) |  |
| Changes from baseline by season^&^ | | | | | | | | | | | | |
| warm season |  | -1.1±11.1 | -3.8±9.7 | 2.12(0.15,4.1) | 0.0354 | <0.001 |  | 1.93(-0.06,3.93) | <0.001 |  | 4.34(0.09,8.58) | 0.033 |
| cold season |  | -0.4±10.9 | 0.2±10.7 | -1.14(-3.04,0.76) | 0.2399 |  |  | -1.26(-3.18,0.65) |  |  | 0.27(-3.67,4.21) |  |

# The overall intervention effect on blood pressure was tested by a linear mixed model for repeated measures, accounting for clustering at the facility level and adjusting for the baseline value.

* To explore the potential interaction between intervention and time of visit, we included intervention, time of visit and the interaction term of intervention*time of visit in the linear mixed model, where the time of visit was treated as nominal variable.

& To explore the potential interaction between intervention and season, we included intervention, time of visit, season of the visit and the interaction term of intervention*season of visit in the linear mixed model. The warm season includes time span from Aril 1 to September 30 and the cold season includes the rest time of the year.

There were some missing data for blood pressure in each visit: N = 116 for 6-month, N = 155 for 12-month, N = 220 for 18-month, N = 230 for 24-month, due to deaths, checking outs, dropping outs or temporary leave

# **Table S2-2 Changes in systolic and diastolic blood pressure in PR versus NR of study salt, in 1195 participants (Per protocol analysis) and participants by addition of extra salt from outside of study in per-protocol analysis**

| **Variables** |  | **PR** | **NR** | **All participants(n=1195)** | | |  | **Not adding extra salt(n=1101)** | |  | **Adding extra salt(n=94)** | |
| --- | --- | --- | --- | --- | --- | --- | --- | --- | --- | --- | --- | --- |
|  |  |  |  | **Difference** | **p value** | **p for interaction** |  | **Difference** | **p for interaction** |  | **Difference** | **p for interaction** |
|  |  | **n = 641** | **n = 554** |  |  |  |  |  |  |  |  |  |
| **Systolic blood pressure** | | | | | | | | | | | | |
| Changes from baseline by visit* | | | | | | | | | | | | |
| 6-month |  | -3.1±20.8 | -2.7±19.6 | 0.74(-1.62,3.1) | 0.5401 | 0.051 |  | 0.02(-2.44,2.49) | 0.066 |  | 8.53(0.36,16.7) | 0.282 |
| 12-month |  | 0.9±21 | 1.3±20.2 | -0.87(-3.25,1.52) | 0.4759 |  |  | -1.17(-3.66,1.33) |  |  | 2.85(-5.42,11.12) |  |
| 18-month |  | -5.1±20.5 | -5.6±19.9 | 0.61(-2.03,3.24) | 0.653 |  |  | 0.03(-2.74,2.8) |  |  | 6.52(-2.24,15.28) |  |
| 24-month |  | -4±20.5 | -1±20.4 | -2.91(-5.46,-0.36) | 0.0255 |  |  | -3.48(-6.17,-0.8) |  |  | 2.87(-5.35,11.1) |  |
| Changes from baseline by season^&^ | | | | | | | | | | | | |
| warm season |  | -4.5±20.1 | -6.9±20 | 2.4(-1.33,6.14) | 0.2074 | < 0.001 |  | 2.03(-1.75,5.81) | <0.001 |  | 7.72(-0.13,15.58) | 0.058 |
| cold season |  | -1.6±21.1 | 1±20 | -2.06(-5.69,1.57) | 0.2658 |  |  | -2.35(-6.01,1.31) |  |  | 1.43(-5.86,8.73) |  |
| **Diastolic blood pressure** | | | | | | | | | | | | |
| Changes from baseline by visit* | | | | | | | | | | | | |
| 6-month |  | -0.8±11.4 | -2.4±10.3 | 0.93(-0.49,2.36) | 0.1997 | <0.001 |  | 0.51(-0.99,2.01) | 0.004 |  | 5.56(1.14,9.99) | 0.032 |
| 12-month |  | 1.6±11.2 | 0.5±10.5 | 0.6(-0.85,2.04) | 0.4174 |  |  | 0.34(-1.18,1.86) |  |  | 3.62(-0.86,8.09) |  |
| 18-month |  | -2±10.9 | -4±10.3 | 1.32(-0.27,2.91) | 0.1047 |  |  | 0.98(-0.7,2.67) |  |  | 4.92(0.18,9.67) |  |
| 24-month |  | -1.3±11 | 0.6±11.2 | -2.05(-3.59,-0.51) | 0.0091 |  |  | -2.22(-3.85,-0.58) |  |  | 0(-4.46,4.45) |  |
| Changes from baseline by season^&^ | | | | | | | | | | | | |
| warm season |  | -1.1±11.1 | -3.1±13.9 | 1.19(-0.64,3.02) | 0.2014 | <0.001 |  | 0.91(-0.95,2.77) | 0.002 |  | 4.27(0.21,8.34) | 0.015 |
| cold season |  | -0.2±11.3 | 0.5±11.1 | -1.13(-2.88,0.61) | 0.2025 |  |  | -1.22(-2.98,0.55) |  |  | -0.21(-3.95,3.53) |  |

# The overall intervention effect on blood pressure was tested by a linear mixed model for repeated measures, accounting for clustering at the facility level and adjusting for the baseline value.

* To explore the potential interaction between intervention and time of visit, we included intervention, time of visit and the interaction term of intervention*time of visit in the linear mixed model, where the time of visit was treated as nominal variable.

& To explore the potential interaction between intervention and season, we included intervention, time of visit, season of the visit and the interaction term of intervention*season of visit in the linear mixed model. The warm season includes time span from Aril 1 to September 30 and the cold season includes the rest time of the year.

There were some missing data for blood pressure in each visit: N = 310 for 6-month, N = 175 for 12-month, N = 412 for 18-month, N = 342 for 24-month, due to deaths, checking outs, dropping outs or temporary leave

# **Table S2-3 Age, sex- and center-adjusted changes in systolic and diastolic blood pressure in PR versus NR of study salt, in 1219 participants and participants by addition of extra salt from outside of study, adjustment for age, sex and region**

| **Variables** |  | **PR** | **NR** | **All participants(n=1219)** | | |  | **Not adding extra salt(n=1124)** | |  | **Adding extra salt(n=95)** | |
| --- | --- | --- | --- | --- | --- | --- | --- | --- | --- | --- | --- | --- |
|  |  |  |  | **Difference** | **p value** | **p for interaction** |  | **Difference** | **p for interaction** |  | **Difference** | **p for interaction** |
|  |  | **n = 645** | **n = 574** |  |  |  |  |  |  |  |  |  |
| **Systolic blood pressure** | | | | | | | | | | | | |
| Changes from baseline by visit* | | | | | | | | | | | | |
| 6-month |  | -3.1±20.8 | -3.1±19.6 | 0.97(-1.37,3.31) | 0.417 | 0.014 |  | 0.32(-2.12,2.76) | 0.025 |  | 8(-0.15,16.15) | 0.277 |
| 12-month |  | 0.8±21.1 | 1±20.3 | -0.72(-3.09,1.65) | 0.5509 |  |  | -0.97(-3.44,1.5) |  |  | 2.52(-5.76,10.8) |  |
| 18-month |  | -5.1±20.5 | -5.9±19.8 | 0.97(-1.64,3.59) | 0.4666 |  |  | 0.39(-2.36,3.13) |  |  | 6.91(-1.81,15.63) |  |
| 24-month |  | -4±20.5 | -0.7±20.6 | -3.18(-5.72,-0.64) | 0.0141 |  |  | -3.7(-6.37,-1.03) |  |  | 2.04(-6.17,10.24) |  |
| Changes from baseline by season^&^ | | | | | | | | | | | | |
| warm season |  | -4.4±18.1 | -6.9±18.2 | 2.32(-1.36,5.99) | 0.2168 | < 0.001 |  | 1.99(-1.71,5.69) | <0.001 |  | 7.19(-0.65,15.02) | 0.049 |
| cold season |  | -1.1±19.9 | 0.5±19 | -2.42(-5.99,1.15) | 0.1844 |  |  | -2.64(-6.23,0.95) |  |  | 0.66(-6.63,7.96) |  |
| **Diastolic blood pressure** | | | | | | | | | | | | |
| Changes from baseline by visit* | | | | | | | | | | | | |
| 6-month |  | -0.8±11.4 | -2.5±10.2 | 1.02(-0.39,2.43) | 0.1544 | <0.001 |  | 0.63(-0.85,2.12) | <0.001 |  | 5.25(0.84,9.66) | 0.027 |
| 12-month |  | 1.5±11.3 | 0.5±10.6 | 0.56(-0.86,1.99) | 0.4366 |  |  | 0.33(-1.17,1.83) |  |  | 3.37(-1.11,7.84) |  |
| 18-month |  | -2.0±10.9 | -4.0±10.2 | 1.48(-0.09,3.05) | 0.0649 |  |  | 1.15(-0.51,2.82) |  |  | 4.94(0.23,9.66) |  |
| 24-month |  | -1.3±11.0 | 0.8±11.3 | -2.29(-3.82,-0.76) | 0.0033 |  |  | -2.43(-4.05,-0.81) |  |  | -0.51(-4.94,3.93) |  |
| Changes from baseline by season^&^ | | | | | | | | | | | | |
| warm season |  | -1.1±9.9 | -3.9±8.6 | 1.57(-0.19,3.33) | 0.0803 | <0.001 |  | 1.33(-0.48,3.13) | <0.001 |  | 4.74(0.79,8.69) | 0.008 |
| cold season |  | 0.3±10.5 | 0.7±9.9 | -1.01(-2.68,0.67) | 0.2393 |  |  | -1.05(-2.76,0.66) |  |  | -0.11(-3.75,3.53) |  |

# The overall intervention effect on blood pressure was tested by a linear mixed model for repeated measures, accounting for clustering at the facility level and adjusting for the baseline value.

* To explore the potential interaction between intervention and time of visit, we included intervention, time of visit and the interaction term of intervention*time of visit in the linear mixed model, where the time of visit was treated as nominal variable.

& To explore the potential interaction between intervention and season, we included intervention, time of visit, season of the visit and the interaction term of intervention*season of visit in the linear mixed model. The warm season includes time span from Aril 1 to September 30 and the cold season includes the rest time of the year.

There were some missing data for blood pressure in each visit: N = 316 for 6-month, N = 178 for 12-month, N = 420 for 18-month, N = 355 for 24-month, due to deaths, checking outs, dropping outs or temporary leave

# **Table S2-4 Changes in systolic and diastolic blood pressure in PR versus NR of study salt in analysis with imputed follow-up measurements**

| **Variables** |  | **PR** | **NR** | **All participants(n=1612)** | |  | **Not adding extra salt(n=1416)** |  | **Adding extra salt(n=196)** |
| --- | --- | --- | --- | --- | --- | --- | --- | --- | --- |
|  |  |  |  | **Difference** | **p value** |  | **Difference** |  | **Difference** |
|  |  | **n = 843** | **n = 769** |  |  |  |  |  |  |
| **Systolic blood pressure** | | | | | | | | | |
| Changes from baseline by visit* | | | | | | | | | |
| 6-month |  | -6.7±22.3 | -8.0±21.6 | 1.33(-2.19,4.86) | 0.4512 |  | 0.84(-2.55,4.23) |  | -0.2(-8.65,8.26) |
| 12-month |  | 1.5±21.7 | 2.3±21.0 | -0.79(-3.52,1.94) | 0.5682 |  | -0.84(-3.8,2.11) |  | 5.33(-3.81,14.46) |
| 18-month |  | -11.0±22.7 | -12.6±22.7 | 1.57(-1.05,4.2) | 0.2387 |  | 1.04(-1.93,4.02) |  | -0.85(-8.68,6.97) |
| 24-month |  | -3.4±20.5 | -0.8±20.8 | -2.67(-5.28,-0.05) | 0.0458 |  | -2.91(-5.73,-0.09) |  | 4.78(-4.77,14.32) |
| Changes from baseline by season^&^ | | | | | | | | | |
| warm season |  | -9.7±22.8 | -12.6±22.1 | 1.51(-2.39,5.41) | 0.4468 |  | 1.46(-3.29,6.2) |  | 4.28(-4.35,12.92) |
| cold season |  | -1.3±21.3 | 1.4±20.7 | -3.11(-6.89,0.67) | 0.1072 |  | -3.02(-7.64,1.6) |  | -1.38(-8.99,6.22) |
| **Diastolic blood pressure** | | | | | | | | | |
| Changes from baseline by visit* | | | | | | | | | |
| 6-month |  | -0.9±11.6 | -1.8±11.4 | 0.94(-1.06,2.94) | 0.3477 |  | 0.76(-1.22,2.74) |  | 2.26(-2.56,7.08) |
| 12-month |  | 1.6±11.4 | 1.7±11.4 | -0.10(-1.66,1.46) | 0.897 |  | -0.21(-1.88,1.45) |  | 0.77(-3.73,5.28) |
| 18-month |  | -3.0±11.1 | -3.9±11.0 | 0.84(-0.69,2.37) | 0.2806 |  | 0.62(-1.01,2.25) |  | 2.38(-2.05,6.8) |
| 24-month |  | -1.0±11.1 | 0.8±11.4 | -1.87(-3.16,-0.57) | 0.0049 |  | -1.97(-3.44,-0.5) |  | -1.11(-5.1,2.88) |
| Changes from baseline by season^&^ | | | | | | | | | |
| warm season |  | -1.8±11.5 | -3.2±11.2 | 1.32(-0.57,3.22) | 0.1705 |  | 1.09(-1.03,3.2) |  | 1.96(-1.77,5.69) |
| cold season |  | -0.1±11.4 | 1.1±11.5 | -1.08(-2.88,0.72) | 0.2387 |  | -1.25(-3.28,0.77) |  | -0.88(-4.63,2.86) |

# The overall intervention effect on blood pressure was tested by a linear mixed model for repeated measures, accounting for clustering at the facility level and adjusting for the baseline value.

* To explore the potential interaction between intervention and time of visit, we included intervention, time of visit and the interaction term of intervention*time of visit in the linear mixed model, where the time of visit was treated as nominal variable.

& To explore the potential interaction between intervention and season, we included intervention, time of visit, season of the visit and the interaction term of intervention*season of visit in the linear mixed model. The warm season includes time span from Aril 1 to September 30 and the cold season includes the rest time of the year.

Multiple imputation by full conditional specification was performed to impute the missing data of blood pressure at follow-up visits. The imputation model includes SBP, DBP and pulse at baseline, 6 months, 12 months and 18 months, as well as residential facilities, center, age, sex and adding salt at 24-month or not.

# **Table S3.** **Association of participants’ reporting on food taste with personal-addition of out-of-study salt at 24-month follow up (N=811)**

| Reporting on food taste | N | Salt addition, n(%) | Odds ratio (95%CI) | p value# |
| --- | --- | --- | --- | --- |
| Bland | 144 | 43 (29.9) | referent | < 0.001 |
| Neutral | 635 | 51 (8.0) | 0.19(0.11,0.31) |  |
| Salty | 32 | 1 (3.1) | 0.07(0.01,0.54) |  |

# Generalized linear mixed model accounting for cluster effect at the facility level.

# **Table S4. Examples of variation between facilities on BP, urinary sodium, saltiness and salt addition**

To help better understand our study results, we did case analysis of the intervention effects on blood pressure, 24-hour sodium excretion, participants’ perception of food saltiness, as well as participant’s self-addition of out-of-study salt. We put these data together, facility by facility, for all facilities that had at least 20 participants measured blood pressure and at least 10 participants measured 24-hour urinary sodium excretion.

We demonstrated the information of four typical facilities as below, according to their randomized group and quality of implementation.

1. A facility assigned to **PR with usual salt** (#117). Data on different outcomes were coherent internally and indicated that the intervention was implemented from the very beginning (even before the baseline) and throughout the whole study period (N = 44 at baseline and N = 24 at 24-month with blood pressure measurements)


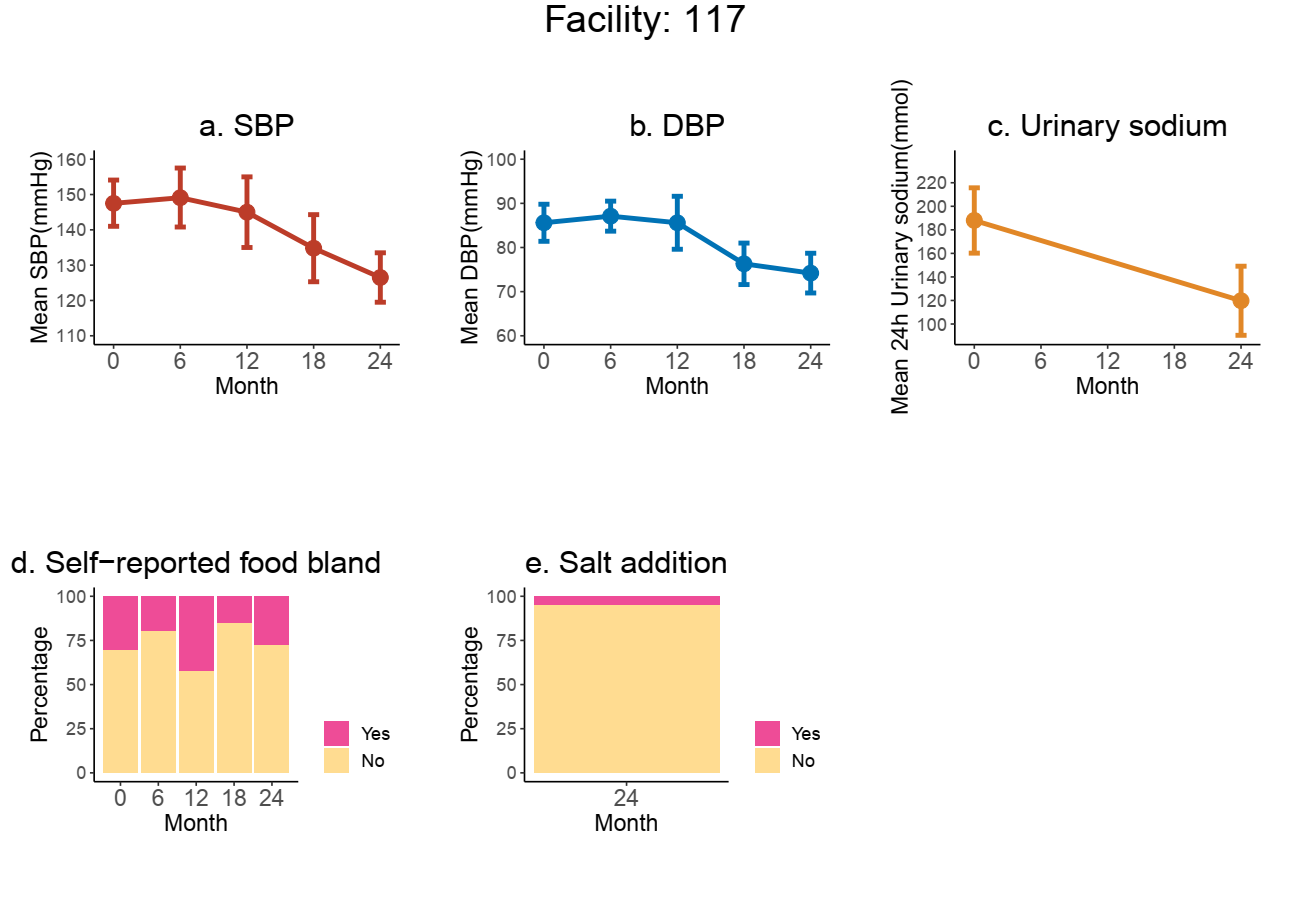


1. A facility assigned to **PR with usual salt** (#307). Data on different outcomes were coherent internally and indicated that the intervention was not implemented well throughout the whole study period (N = 79 at baseline and 42 at 24-month with blood pressure measurements)


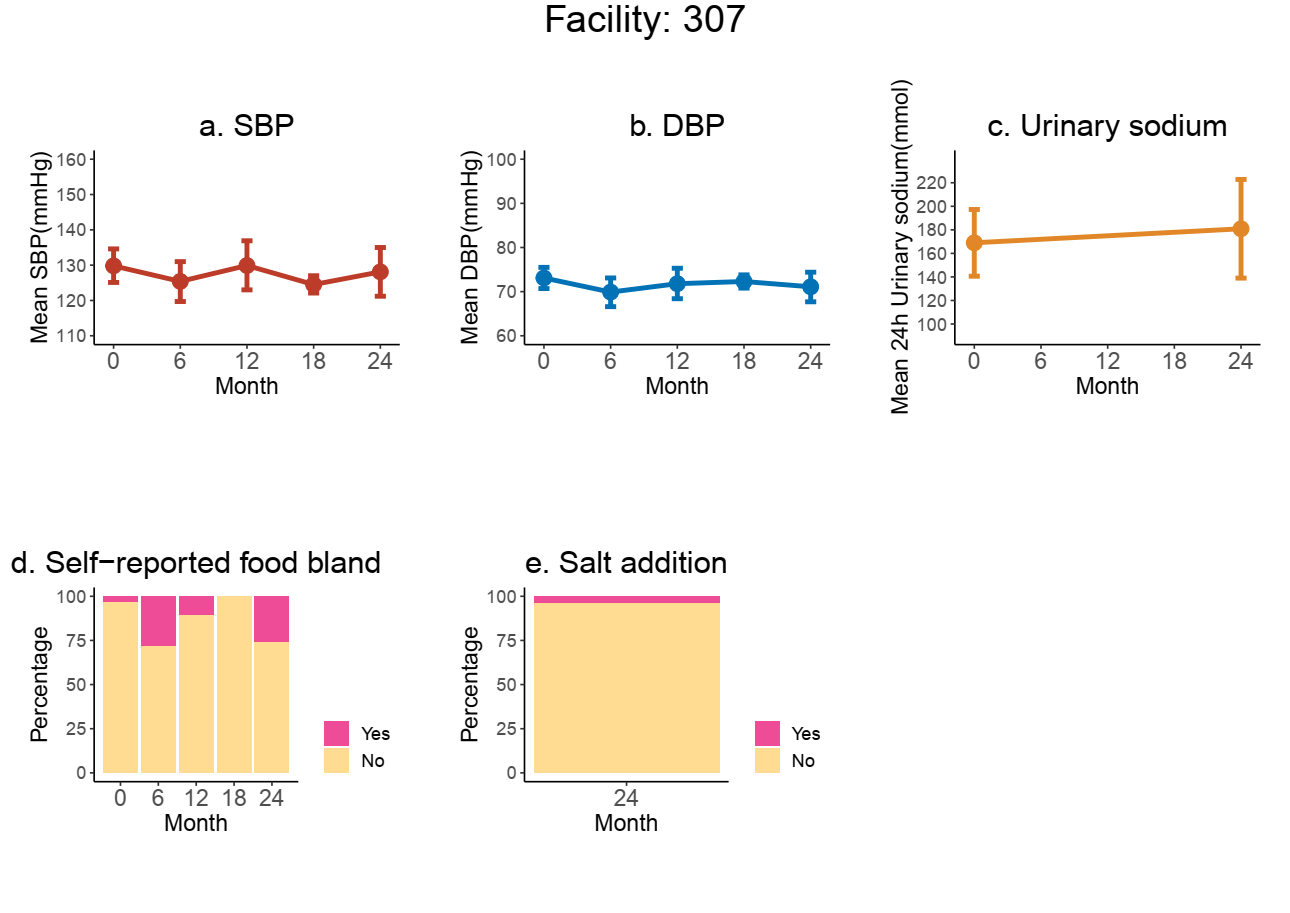


1. A facility assigned to **NR with usual salt** (#109). Data on different outcomes were coherent internally and indicated that the facility was not compliant to the assigned intervention and started to restrict the salt supply from the very beginning (even before the baseline) and throughout the whole study period. (N = 39 at baseline and 36 at 24-month with blood pressure measurements)


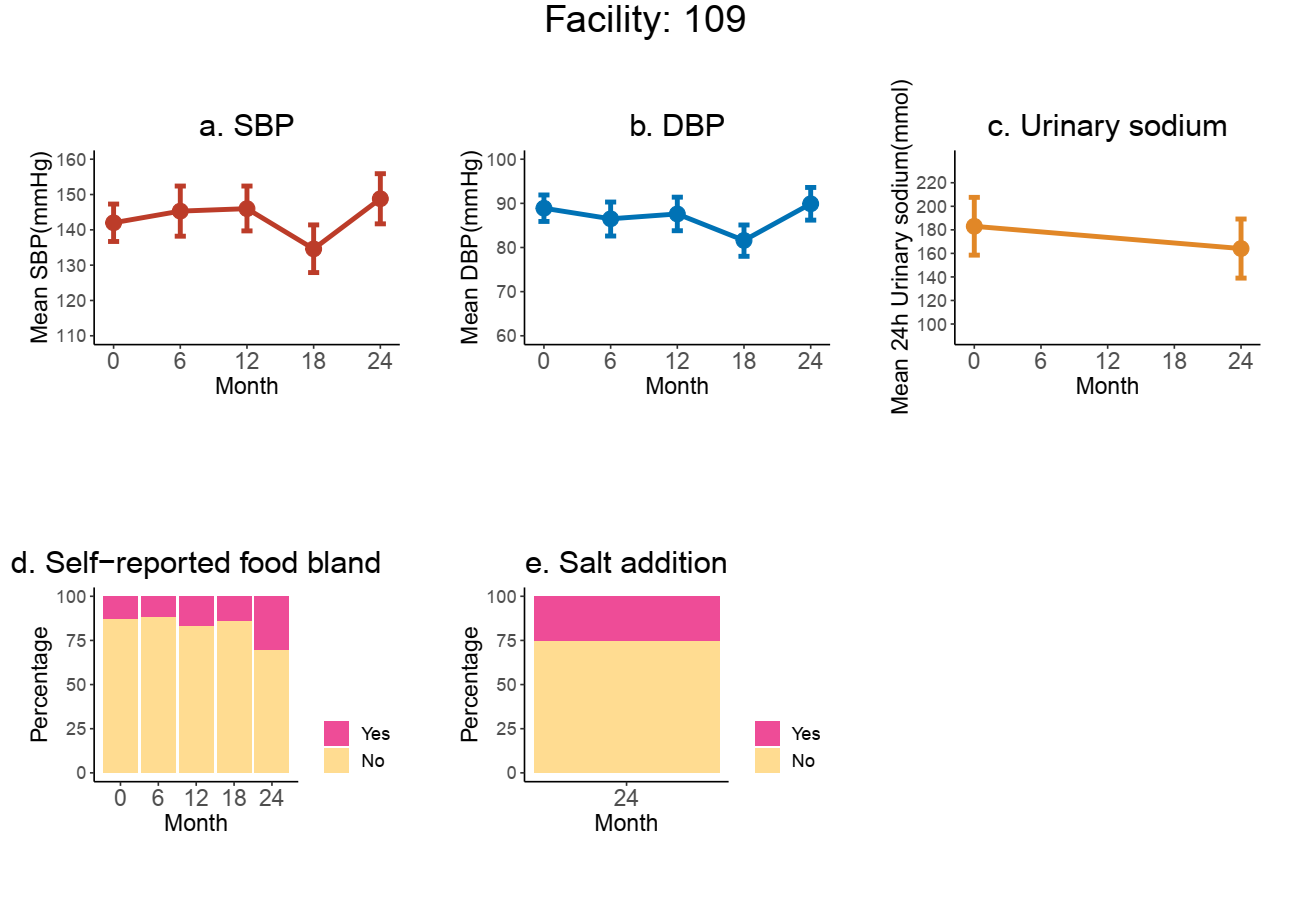


1. A facility assigned to **NR with usual salt** (#411). Data on different outcomes were coherent internally and indicated that the facility was compliant to the intervention assigned by the study throughout the whole study period. (N = 27 at baseline and 22 at 24-month with blood pressure measurements)


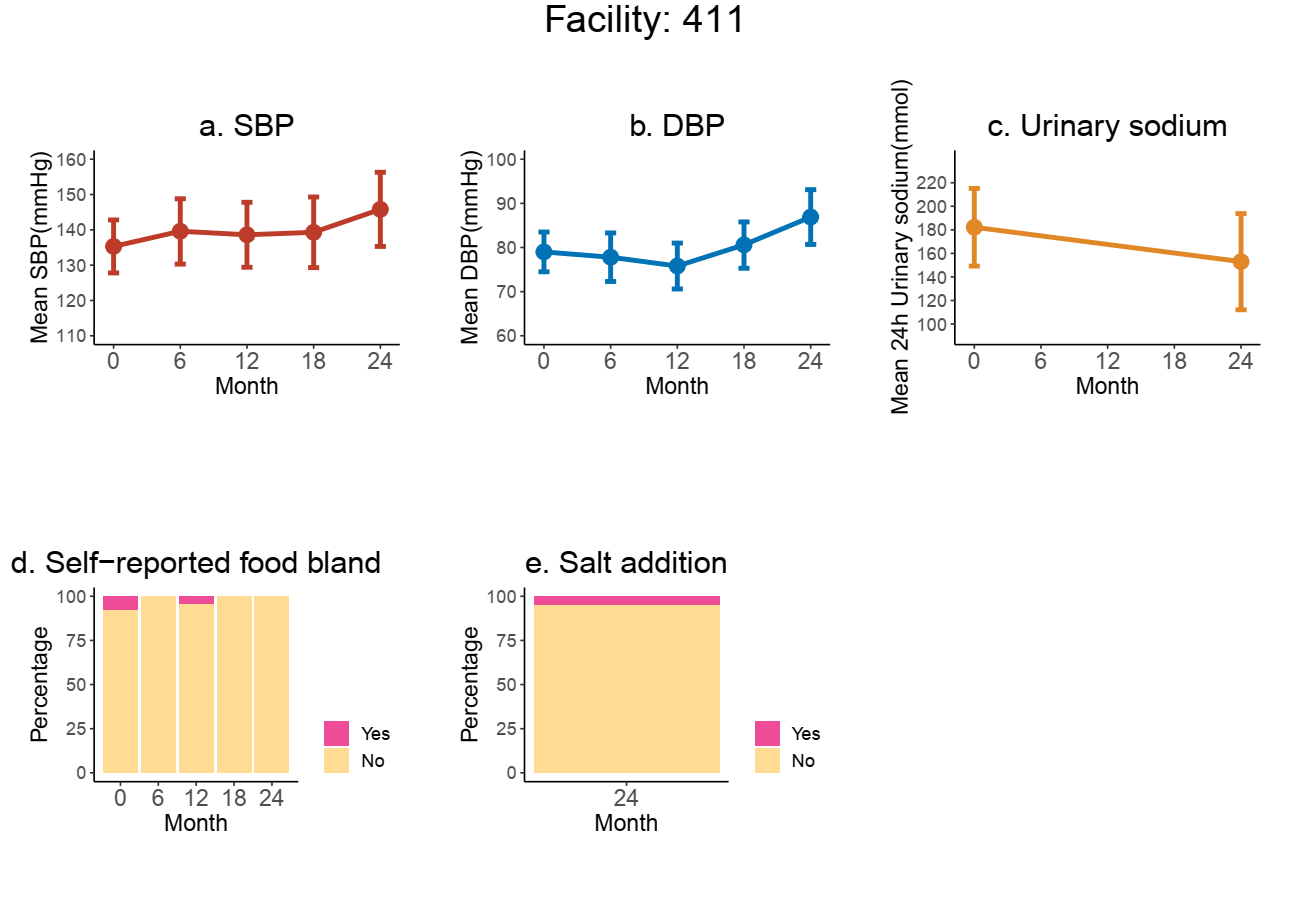

Supplement: Supplementary file 1 — Additional file 1: Table S1. Evaluation of possible bias from missing data in main results in DECIDE-Salt trial. Table S1-1. Comparison between intervention groups in percentages of participants actually analyzed for the assessment of effect on blood pressure and 24-hr urine electrolytes. Table S1-2. Comparisons between participants analyzed and not analyzed in baseline characteristics, among 1612 eligible participants for the assessment of effect on blood pressure. Table S1-3. Comparisons between participants analyzed and not analyzed in baseline characteristics, among 1612 eligible participants for the assessment of effect on urinary sodium. Table S1-4. Comparisons between participants analyzed and not analyzed in baseline characteristics, among 1612 eligible participants for the assessment of food saltiness. Table S2. Secondary analysis on effect on blood pressure. Table S2-1. Changes in systolic and diastolic blood pressure in PR versus NR of study salt, in participants excluding Xi’an (N = 1019) and participants by addition of extra salt from outside of study. Table S2-2. Changes in systolic and diastolic blood pressure in PR versus NR of study salt, in 1195 participants (Per protocol analysis) and participants by addition of extra salt from outside of study in per-protocol analysis. Table S2-3. Age, sex- and center-adjusted changes in systolic and diastolic blood pressure in PR versus NR of study salt, in 1219 participants and participants by addition of extra salt from outside of study, adjustment for age, sex and region. Table S2-4. Changes in systolic and diastolic blood pressure in PR versus NR of study salt in analysis with imputed follow-up measurements. Table S3. Association of participants’ reporting on food taste with personal-addition of out-of-study salt at 24-month follow up. Table S4. Examples of variation between facilities on BP, urinary sodium, saltiness and salt addition. [file 12916_2023_3130_MOESM1_ESM.docx]
