# Supplementary material for: Experience with 2 years’ intervention to progressively reduce salt supply to kitchens in elderly care facilities—challenges and further research: post hoc analysis of the DECIDE-Salt randomized clinical trial
Source: BMC Med. 2023 Nov 3;21:416. doi: 10.1186/s12916-023-03130-z (PMC10623877; doi:10.1186/s12916-023-03130-z)
Supplement: Supplementary file 2 — Additional file 2. Supplementary Appendix on statistical methods. [file 12916_2023_3130_MOESM2_ESM.docx]

**Supplementary Appendix on statistical methods**

1. **Statistical model used for primary analysis on effect on blood pressure**

The original model in the SAP assumed that the intervention effect was constant over 2 years. That may be correct for the intervention with salt substitute, which take effect in few weeks, but should not be appropriate for the intervention with stepwise progressive restriction of salt supply, for which the effect would be increasing or vary by duration of the intervention even if it is effective. Thus, we replaced the original model with a modified model to better reflect the nature of the intervention.

**Original model in the SAP**

A pre-specified linear mixed effects model will be performed to model repeated measures of SBP of the same individual over time. The model will include intervention status and baseline SBP as fixed effects, as well as cluster and time as random effects. Separated models will be built for the intervention. The model would be:

$$y_{ijt}= \beta_{0}+ \mu_{1i}+ \mu_{2j}+\beta_{1}x_{ij}+\beta_{2}c_{ij}+\beta_{3}t+ e_{ijt}$$

$$e_{ijt}\sim N(0,\sigma_{e}^{2})$$

$$\mu_{1i}\sim N\left( 0,\sigma_{\mu1}^{2} \right)$$

$$\mu_{2j}\sim N\left( 0,\sigma_{\mu2}^{2} \right)$$

Where *i* represents the i^th^ subjects, *j* represents the j^th^ cluster (facility), *t* represents time point t, $y_{ijt}$ is the SBP measured for subject *i* in cluster *j* at time *t* (follow-up month), baseline SBP will not be included in $y_{ijt}$; $\beta_{0}$ is the mean outcome in the control group at baseline; $\mu_{1i}$ is an individual random effect to allow for multiple repeats per person; $\mu_{2j}$ is a cluster random effect on baseline SBP to allow for between-cluster heterogeneity ; $x_{ij}$ *,* the predictor of interest is an indicator of the intervention for subject i in cluster j (1 indicates receive intervention while 0 indicates not receive intervention); *β_1_* , the coefficient of interest , is the effect coefficient of intervention; $c_{ij}$ is the baseline SBP measured for subject i in cluster j ; *e_ijt_* is the random error for the measurement of subject i in cluster j at time t.

**Modified statistical model**

For the analysis of the effect of intervention on SBP, the linear mixed model was modified to allow the intervention effect vary from baseline to each follow-up visit, i.e. adding the interaction term. That is, we included group (intervention, control), time (baseline and all follow-up visits), and time×group interaction. The group was treated as fixed effect, while the cluster and subject were treated as random effects. The model was:

$$y_{ijt}= \beta_{0}+ \mu_{1i}+ \mu_{2j}+\beta_{1}x_{ij}+\beta_{2}t +\beta_{3}x_{ij}*t+ e_{ijt}$$

$$e_{ijt}\sim N(0,\sigma_{e}^{2})$$

$$\mu_{1i}\sim N\left( 0,\sigma_{\mu1}^{2} \right)$$

$$\mu_{2j}\sim N\left( 0,\sigma_{\mu2}^{2} \right)$$

Where $i$ represents the i^th^ subjects, $j$ represents the j^th^ cluster (facility), $t$ represents time point t, $y_{ijt}$ is the SBP measured for subject *i* in cluster *j* at time *t* (0 for baseline and follow-up month for follow-up visits); $\beta_{0}$ is the mean SBP in the control group at baseline; $\mu_{1i}$ is an individual random effect to allow for multiple repeats per person; $\mu_{2j}$ is a cluster random effect on baseline SBP to allow for between-cluster heterogeneity ; $x_{ij}$ is an indicator of the group for subject i in cluster j (1 indicates receive intervention while 0 indicates not receive intervention; $t$ is an indicator of time for each visit (0 for baseline and follow-up month for follow-up visits); $x_{ij}*t$ , the predictor of interest, is the interaction term of time by group; $\beta_{3}$ is the effect coefficient of the interaction term; $e_{ijt}$ is the random error for the measurement of subject i in cluster j at time t.

The analysis on the effect of intervention on DBP and urinary sodium used the same model as above.

1. **Models used for secondary analysis on effect on blood pressure**

The post-hoc analysis explored the possible variation in intervention effect by season using a linear mixed model that allows the effect of intervention vary by seasons. The model included group (intervention, control), baseline SBP, time (all follow-up visits), season(cold, warm) and season×group interaction. The group, baseline SBP and season were treated as fixed effect, while the cluster and subject were treated as random effects. The model was:

$$y_{ijt}= \beta_{0}+ \mu_{1i}+ \mu_{2j}+\beta_{1}x_{ij}+\beta_{2}c_{ij}+\beta_{3}t++\beta_{4}S_{ij}+ \beta_{5}S_{ij}* x_{ij}+ e_{ijt}$$

$$e_{ijt}\sim N(0,\sigma_{e}^{2})$$

$$\mu_{1i}\sim N\left( 0,\sigma_{\mu1}^{2} \right)$$

$$\mu_{2j}\sim N\left( 0,\sigma_{\mu2}^{2} \right)$$

Where *i* represents the i^th^ subjects, *j* represents the j^th^ cluster (facility), *t* represents time point t, $y_{ijt}$ is the SBP measured for subject *i* in cluster *j* at time *t* (follow-up month), baseline SBP will not be included in $y_{ijt}$; $\beta_{0}$ is the mean outcome in the control group at baseline; $\mu_{1i}$ is an individual random effect to allow for multiple repeats per person; $\mu_{2j}$ is a cluster random effect on baseline SBP to allow for between-cluster heterogeneity ; $x_{ij}$ is an indicator of the group for subject i in cluster j (1 indicates receive intervention while 0 indicates not receive intervention); $c_{ij}$ is the baseline SBP measured for subject i in cluster j ; $t$ is an indicator of time for each visit (follow-up month for follow-up visits); $S_{ij}$ is an indicator of the season for subject i in cluster j (1 indicates warm season while 2 indicates cold season); $S_{ij}* x_{ij}$, the predictor of interest, is the interaction term of season by group; $\beta_{5}$ is the effect coefficient of the interaction term; *e_ijt_* is the random error for the measurement of subject i in cluster j at time t.

The analysis on the effect of intervention on DBP used the same model as above.

1. **Models for imputing the missing values of follow up SBP and DBP**

Multiple imputation by full conditional specification was performed to impute the missing data of blood pressure at follow-up visits. The imputation model includes SBP, DBP and pulse at baseline, 6 months, 12 months and 18 months, as well as residential facilities, center, age, sex and adding salt at 24-month or not.
